# Primary seronegative intermuscular hydatidosis in a child, an infection that should be approached like a tumor: a case report and review of the literature

**DOI:** 10.1186/s13256-024-04405-6

**Published:** 2024-03-08

**Authors:** Reza Tavakoli Darestani, Gholamhossein Kazemian, Sina Afzal, Mojtaba Baroutkoub, Mahdi Aghaalikhani, Farzad Amouzadeh Omrani

**Affiliations:** Department of Orthopedic Surgery, School of Medicine, Shahid Beheshti University of Medical Sciences, Tehran, Iran

**Keywords:** Hydatid cyst, Musculoskeletal, *Echinococcus granulosus*, Tumor, Case report

## Abstract

**Introduction:**

Intermuscular hydatid cyst is one of the rarest types of hydatid cyst, and as far as we know, only nine cases were reported in the literature before this study.

**Case presentation:**

We present a 10-year-old Iranian child with an intermuscular cystic mass in the medial-distal thigh. Despite the typical imaging findings, the patient's serological and hematological tests were negative for hydatid cyst. The cyst underwent wide excision accompanied by neoadjuvant and adjuvant chemotherapy with Albendazole. No evidence of recurrence was detected during the one-year follow-up.

**Conclusion:**

Hydatid cysts should always be considered in the differential diagnosis of soft tissue cystic masses in endemic areas, and aspiration or drainage should be avoided as much as possible, even when serological tests are negative and imaging is non-diagnostic. In cases where the diagnosis of a hydatid cyst has been confirmed before the surgery, it is recommended to approach the cyst, like a tumor with chemotherapy using Albendazole both before and after wide cyst excision.

## Introduction

Hydatidosis is a zoonosis in which canines, ovine animals, and humans serve as the definitive, intermediate, and incidental hosts of a parasite called Echinococcus, respectively. Following the accidental entry of this helminth's ova into the human body via the oral-fecal route, the larva can invade anywhere in the body, most notably the liver and lungs [1].

Musculoskeletal involvement is infrequent, accounting for less than 5% of all cases, even in endemic areas such as the Middle East [2]. The muscles are less susceptible to hydatid infestation than the skeletal system, which can be linked to a combination of factors such as muscular contractility, oxygen insufficiency, and lactate excess. Most cases of muscular hydatidosis are intra-muscular cysts secondary to liver and lung cysts, and primary muscle involvement, especially inter-muscular cysts is very rare [3]. Extremity infections mainly occur in limbs proximally, like the groin and thigh, due to less muscle contractility and better blood supply and are less likely to happen distally [4].

Due to oral-fecal transmission, children are more susceptible to infection; however, due to the cyst’s slow development rate, symptoms mostly manifest in adults [5, 6]. Therefore, just 10–20% of all hydatid cysts involve under-16-year-old patients [7]. This study aims to introduce a rare case of an inter-muscular hydatid cyst in a 10-year-old girl, presented as a gradually-growing mass around the knee.

## Case presentation

A 10-year-old Iranian girl was referred for evaluation of a large mass in the medial aspect of the distal right thigh. The mass was found at age five and diagnosed as lipoma by a pediatrician who cautioned the parents to return if it grew faster or caused new symptoms. The patient returned after 5 years with an enlarged mass. The mass was painless, mobile, well-defined, non-pulsatile, non-inflamed, and 5 cm wide in diameter. She was a student and lived with her family in an urban area. They had no previous medical issues and denied smoking and substance abuse.

She had no abdominal pain or discomfort, no chest pain or cough. There was no history of weight loss, hyperhidrosis, trauma, walking issues, discharge, or lesions on other sites. The patient's vital signs were normal, she was afebrile, and the neuro-vascular assessment was normal.

**Fig. 1:**
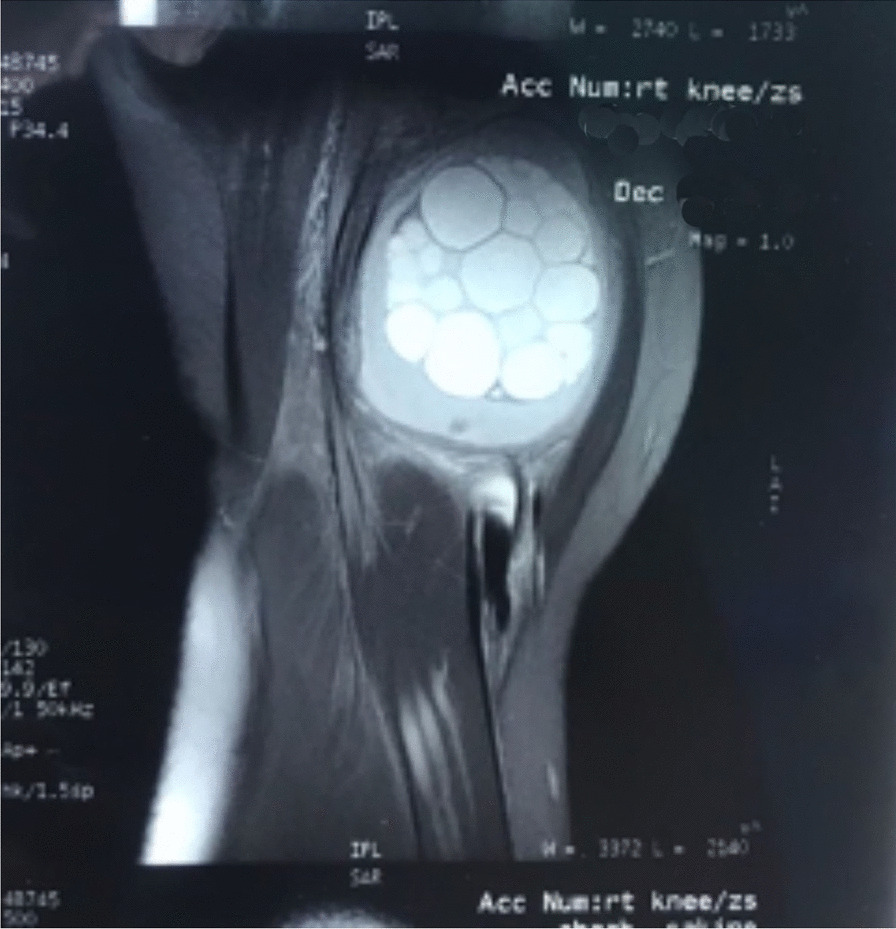
Sagittal T2 weighted knee MRI. The presence of multiple hyper signal daughter cysts and a thick pericyst

The ultrasonography evaluation demonstrated an extra-articular multilobular cystic lesion in the right distal thigh, characterized by a thickened wall and collapsible hypoechoic structures. Magnetic resonance imaging (MRI) revealed a multicystic lesion measuring 7 × 7 cm between the Sartorius and vastus medialis muscles. Contents of cysts show high T2 and low T1 signal intensity, and the whole lesion had a thick irregular capsule with distinct borders (Fig. 1). No abnormal enhancement or signs of solid components were seen in post-contrast images. Based on the imaging results, the hydatid cyst and benign cystic soft tissue tumor emerged as the primary differential diagnosis; therefore, additional radiological investigations of various body regions and serological tests were undertaken on the patient.

Despite the lack of exposure to dogs, laboratory studies for hydatid cysts were conducted for her due to MRI characteristics and the hydatidosis endemicity in Iran. All hematologic and serologic tests, including specific Immunoglobulin G against *Echinococcus* *granulosus*, total IgE, eosinophil count, ESR, and CRP, were normal.

**Fig. 2:**
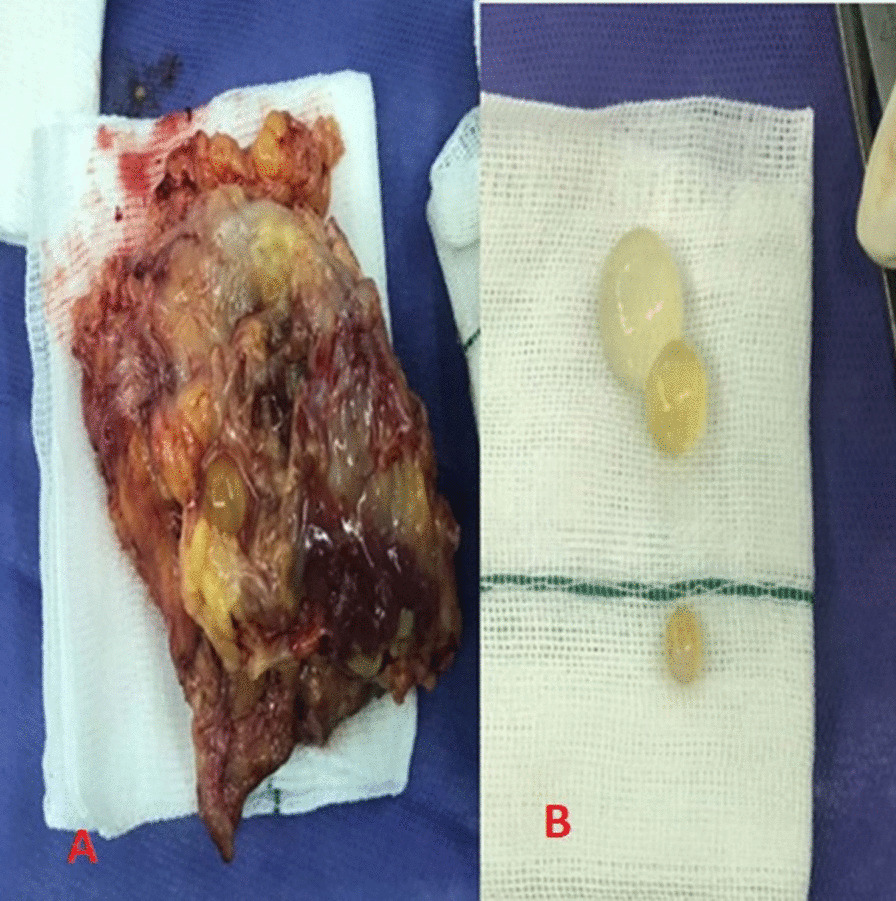
Gross pathology of resected cyst. **A** Excised cyst with a margin of host tissues (negative resection margin). **B** Daughter cysts inside the mother cyst

Considering that one of the primary diagnoses was hydatid cyst, we administered Albendazole 15 mg/kg/day for 8 weeks preoperatively based on the infectious diseases service consultation. We prepared the patient for a wide excisional biopsy. Under general anesthesia and via medial approach, using an elliptical incision and preserving the infrapatellar branch of the saphenous nerve, an oval cyst with a diameter of about 7 cm was found after opening the fascia between the vastus medialis and Sartorius muscles. The cyst was carefully excised with about 1cm margin without damaging the capsule (Fig. 2). After dissecting the cyst and observing the germinal membrane and daughter cysts, the diagnosis of a hydatid cyst was confirmed. For more caution, gowns, gloves, and surgical sets were changed, and adding the suture, the operative field was washed with hypertonic saline. After inserting two subfascial vacuum drains, the fascia was sewn layer by layer.

The wound healed 2 weeks after surgery, and the stitches were removed. The infectious diseases service suggested to continue Albendazole 15 mg/kg/day for 3 months following surgery. In addition to reconfirming the diagnosis of hydatid cyst via histopathology, the results indicated that the protoscolices were non-viable. Following the pathologist’s confirmation of the diagnosis, abdominal and chest computed tomography (CT) scans and MRI of the brain were conducted to rule out multifocal hydatidosis. Twelve months after surgery, imaging studies exhibited no evidence of recurrence.

## Discussion

The word “hydatid” is derived from an ancient Greek word and means “watery vesicle”. However, in medical science, the term "hydatid cyst" refers to the larval stage of a zoonotic infection that invades human hosts incidentally. The pathogen responsible for this disease is *Echinococcus granulosus*. Given that the definitive and intermediate hosts of this parasite are canines and livestock, hydatidosis is predominantly seen in rural areas, and endemic in livestock-rearing regions, such as the Mediterranean and Middle East countries [8]. Hydatid cysts are more prevalent in rural areas but have also been reported in urban settings, like this introduced patient [9].

Hydatidosis can involve any part of body either primary or secondary. In primary cases, the ingested embryos infest anywhere in the body but typically the liver via blood circulation. In secondary cases, protoscolices originated from primary cysts are accountable for the genesis of cysts in other locations of body. Some studies showed that lungs are the most commonly affected organs in children, as they are more elastic and allow cysts to grow more easily [10, 11].

Primary musculoskeletal hydatid cyst usually infects the children, but due to its slow growth rate (1–5 cm/year), it is typically asymptomatic during childhood and is not diagnosed until adulthood, except for subcutaneous cysts which are more frequently diagnosed in children due to their visibility. Consequently, sub-fascial echinococcosis including intermuscular or intramuscular cysts, are less commonly visible and diagnosed in children [7].

Soft tissue hydatid cysts typically present as a painless swelling with varying degrees of mobility, but they may also be detected incidentally without any observable symptoms [9, 12, 13]. Other symptoms such as cellulitis or neuropathy and limb circulatory disorders may occur due to the cyst rupture or compression of the surrounding structures [14–17].

Ultrasonography may be helpful in the diagnosis of the majority of cases with typical characteristics; however, MRI is the imaging modality of choice for the diagnosis and evaluation of musculoskeletal hydatid cysts [18]. Echinococcosis typically is hypersignal in T2 and hyposignal in T1; however, this is not always the case, and it might vary depending on the stage of development of the cyst. Another benefit of MRI, is the diagnosis of daughter cysts within the mother cyst, which is pathognomonic of hydatid cysts [19].

Although positive serological test results can help in confirmation of imaging investigations, but negative results cannot rule out hydatidosis due to their limited sensitivity (less than 30%), especially in extrahepatic cases and children. Hematological studies are also not very helpful in diagnosing the disease, as they are normal in most cases, except for the rare cases with eosinophilia [9, 13, 20, 21]. If preoperative imaging and serological tests do not allow a definitive diagnosis of hydatidosis, a definitive diagnosis is made postoperatively by histopathological visualization of germinal membranes or protoscolices [22].

In such cases, especially in endemic areas, considering that hydatid cyst rupture and spilling of its contents can lead to debilitating and fatal complications such as recurrence of the lesion and anaphylactic shock, biopsy of the lesion should be done in the form of wide excision and other methods including FNA or incisional biopsy are contraindicated [23]. If a hydatid cyst diagnosis is confirmed, the liver, lungs, and brain must be examined to determine whether the primary source is present or the lesion is primary itself [24].

Neoadjuvant therapy with Albendazole at 10–15 mg/kg daily for 4–12 weeks before surgery significantly reduces the risk of disease recurrence and secondary hydatidosis [25, 26]. It also reduces intracystic pressure, making the cyst easier to excise and less likely to rupture during surgery [27]. While there is still no comprehensive agreement regarding adjuvant chemotherapy after surgery, and while many articles recommend drug therapy after cyst excision, other articles believe that in case of complete cyst excision with a margin, there is no need for postoperative drug therapy [21, 25].

In this table, you can see an overview of information on previous reported inter-muscular hydatid cysts (Table 1).

**Table 1:** Characteristics of previously reported cases of intermuscular hydatidosis

Case no	Age	Sex	Location	Serology	Hematology	Procedures	Time of diagnosis	Chemotherapy
1 [28]	18	M	Forearm	Negative	Negative	Excision	Preoperative	NR
2 [29]	16	F	Thigh	Negative	Negative	Excision	Preoperative	Two weeks adjuvant therapy
3 [6]	4	F	Thigh	Negative	Negative	Excision	Preoperative	Ten days neoadjuvant, and 3 months adjuvant therapy
4 [30]	70	F	Thigh	Negative	Negative	FNA + excision	After FNA	Three months adjuvant therapy
5 [14]	48	M	Thigh	Positive		FNA + excision	After FNA	Four months adjuvant therapy
6 [31]	30	M	Arm	Positive	Negative	Excision	Preoperative	Six weeks adjuvant therapy
7 [32]	30	F	Thigh	Positive	Negative	Drainage + excision	After recurrence	Two weeks neoadjuvant and 4 weeks adjuvant therapy
8 [33]	30	M	Scapula	Positive	Eosinophilia	FNA + excision	After FNA	Neoadjuvant and adjuvant therapy (Durations: NR)
9 [34]	65	F	Scapula	Negative	Negative	Excision	Intraoperative	Three months adjuvant therapy

M: Male; F: Female; NR: Not reported

## Conclusion

Intermuscular hydatid cyst is very rare, and in some cases, it may be confused with soft tissue tumors due to non-specific imaging findings and negative serology tests. In endemic areas, hydatid cyst should be considered as one of the differential diagnoses in cases of soft tissue cystic mass with non-specific imaging and laboratory findings. If this disease is suspected, it is recommended to excise the mass without damaging its wall, along with neoadjuvant chemotherapy with Albendazole before surgery. Histopathological findings could provide a definite identification of the cyst's nature, which determines the next course of treatment.

## Data Availability

The material presented in this study are available from the corresponding author on a reasonable request.

## Abbreviations

MRI: Magnetic resonance imaging
CT: Computed tomography
FNA: Fine-needle aspiration
